# Consumption and Infection

**Published:** 1894-04-07

**Authors:** 


### Consumption and Infection.

There seems some reason for believing that men's minds are undergoing a certain rebound from the extreme opinions advanced in certain quarters during the past year or two in regard to the infectious nature of consumption; not that there is any hesitation in accepting the bacillus as the true cause of the disease, but that, as the result of the matter being looked at from many points of view, it is becoming increasingly clear that in estimating its contagiousness or infectiveness from person to person, many other factors besides the mere presence of the bacillus have to be taken into consideration.

Last year Dr. Chaplin, writing in the *Medical Review*, put forward with great force the extreme view that since the disease results from the growth of a parasitic organism, which is being continually scattered far and wide by those who are already suffering from it, these sufferers should be dealt with in the same manner as those who are known to be affected with other infectious disorders. He frankly admitted the difficulties, but maintained that the only logical outcome of our present knowledge was to segregate consumptives, and prevent them from continuing to propagate the disease. Meanwhile the knowledge that the common source of infection was the respiration of dust, consisting partly of the dried sputa of consumptive people, had led to great efforts being made to encourage, and even enforce, the constant use of the spitoon, to prevent in every way the free spitting about which is so common in every place, and especially to dissuade consumptives from spitting into their handkerchiefs and so disseminating the dried sputa, in the form of dust, throughout their dwelling rooms. The matter was largely taken up as a sanitary question by medical officers of health, and in the North of England not only was a great deal of educational work done by the distribution of printed matter, by the encouragement of notification, and by free disinfection by certain municipalities of houses occupied by consumptives, but very stringent measures were proposed, viz., that tuberculosis should be made a notifiable disease, the same as scarlet fever, and that isolation hospitals should be provided by sanitary authorities at the expense of the rates for the isolation of cases of tuberculosis likely to cause infection. It is noteworthy, also, that Dr. Niven, by whom these proposals were put forward, has since been appointed medical officer of health by so large a muncipality as that of Manchester. It was admitted then that tuberculosis was a parasitic, and therefore an infectious disease; that the vehicle by which it was spread was the dried sputum of tuberculous patients, and the conclusion was gradually forcing itself forwards that this source of infection could be effectually controlled by nothing short of the segregation of the consumptives.

Such ideas as these did very well for sanitarians who deal with humanity in bulk, but those who were concerned with the individual, and, taking their cases one by one, considered how such regulations would affect them, were not slow to give expression to the impracticability of attempts to interfere with the liberty of so large a number of men who, during a great portion of what might be termed their period of infectiousness, were fairly useful members of society and quite able to earn their own living. Dr. Kanthack also traversed the whole ground of the contagionists, showing that no amount of proof that a disease was infective was any proof at all that this was the reason of its spread. We know perfectly well that when jam goes mouldy the real cause is an infection by a fungus; but we all know that, when this mould infests a cupboard, the real reason is that the cupboard is damp or the jam defective. It is the nature of the constitution or surroundings, rather than the mere presence of the germ, that makes it spread; and Dr. Kanthack pointed out that the same was true in regard to diseases, and that animals which in their ordinary state are practically immune to certain maladies can be rendered susceptible by merely altering their surroundings. In studying the etiology of consumption then we must in no way be content with the knowledge that it is a microbic disease, and therefore due to an infection, but must search out what are the conditions which make man susceptible to it. For as a starting point there are but small reasons for believing that normal man is susceptible to tuberculosis; not only do large numbers of people, apparently exposed to the same risks as others who die of consumption, entirely escape infection, in which of course tuberculosis only resembles other infectious diseases, but patients who already suffer, who carry the infection constantly within them, may long escape any generalisation of the malady, and may even recover entirely, so little are normal tissues favourable to the growth of this microbe. The results recently obtained by surgeons in the treatment of tubercular peritonitis by laparotomy point in the same direction, showing as they do that so small a matter as the removal of the surrounding fluid may decide the question whether the tubercles shall progress or become obsolescent. This brings one back at once to the older view of the preponderating influence of predisposition. In a book published at the beginning of this year by Dr. Edward Squire on the prevention of consumption, while full credit is given to the bacillus, and all due stress is laid on the importance of careful management of the sputa, and preventing the diffusion of tuberculous dust, the facts are emphasised that the predisposing conditions are those with which we can most effectually deal, and that, among these conditions, general states of health, or departures therefrom, are even more important than the occurrence of local diseases.

It would seem then that we are now able to estimate the various factors involved in the production of consumption. We have (*a*) the bacillus, the *causa causans*, without which all other influences would be inoperative; (*b*) a condition of body, a predisposition, which may be inherited, or may be acquired from the conditions amid which life is passed, of which dampness of soil, dampness of house, want of light, deficient ventilation, uncleanliness, innutritious food, and unhealthy occupations form the chief; (*c*) conditions of defective nutrition consequent on illness; and (*d*) local states of malnutrition resulting from diseases of various organs. In a lecture given lately before the National Health Society attention was drawn to the fact that, although the war against tuberculous dust is only just beginning, the mortality from phthisis has for a long series of years been steadily diminishing. Dividing the years from 1861 to 1885 into periods of five years each, we have the consumption death-rate, per 1,000 living, running a descending scale as follows: 2·5, 2·4, 2·2, 2·0, 1·8; or, putting the matter in a different way, while in the period 1851-60 one-eighth of all deaths were put down to phthisis, the proportion had fallen in 1885 to one-tenth, and in 1890 to one-eleventh, and this as the result of general amelioration of the conditions of life. Spitting cups and efforts to prevent tuberculous dust by keeping the sputa wet are of prime importance, but do not take the place of those hygienic measures which tend to lessen man's predisposition to the disease. For growth depends on soil as much as upon seed.

